# Oral Manifestations of Syphilis: Report of Four Cases

**DOI:** 10.3390/pathogens11060612

**Published:** 2022-05-24

**Authors:** Suné Mulder van Staden, Carl de Villiers, Julandi Alwan, Mpho Moloi, Sibongile Mahlangu

**Affiliations:** 1 Oral Medicine and Periodontology Department, Faculty of Dentistry, The University of the Western Cape, Cape Town 7530, South Africa; devillierscarl@gmail.com (C.d.V.); mphomoloi88@gmail.com (M.M.); drspmahlangu@gmail.com (S.M.); 2 National Health Laboratory Service (NHLS), Tygerberg Academic Laboratories, Tygerberg Hospital, Cape Town 7505, South Africa; julandi.alwan@nhls.ac.za

**Keywords:** syphilis, oral lesions, *Treponema pallidum*, sexually transmitted infections, oral health

## Abstract

Syphilis is an infectious disease caused by *Treponema pallidum.* Syphilis can present with an array of oral manifestations at different stages of disease progression. This article reports on four cases of syphilis with oral manifestations diagnosed by oral health professionals. Case 1: 18-year-old female presented with multiple ulcerations and patches involving the hard palate, uvula, retromolar area, and papillary nodules on the tongue. Case 2: 25-year-old male presented with a solitary, exophytic lesion on the anterior tongue. Case 3: 17-year-old female presented with multiple pigmented macules on the palms of hands and soles of feet, as well as multiple exophytic, sessile, soft tissue masses throughout the oral cavity. Case 4: 14-year-old female presented with a solitary, exophytic, verrucous lesion in the incisive papilla area, as well as multiple, coalescing patches involving the soft palate, uvula, and tonsillar areas. All patients were managed by biopsy and serological investigations. Treatment of syphilis was performed at infectious disease clinics with 2.4 million units (MUs) benzathine benzylpenicillin intramuscular (IM) weekly. Oral health professionals need to have knowledge of the oral manifestations of syphilis to ensure that patients are accurately identified and appropriately treated.

## 1. Introduction

In developing countries such as South Africa, sexually transmitted infections (STIs) are an important public health concern [1], enhanced by Human Immunodeficiency Virus (HIV) transmission, cardiovascular complications and severe neurological disability if not managed appropriately [2].

The dental practice is a unique environment where syphilis could potentially be spread by direct contact with lesions, as well as patient’s fluids—such as blood and saliva [3]. Patients may also present for treatment specifically due to the presence of oral lesions. Oral health professionals (OHPs), e.g., dentists, dental therapists, dental hygienists, and dental specialists, thus play an important role in syphilis recognition and appropriate treatment implementation or referral of patients presenting with oral manifestations of this disease. The aim of this paper is to report on four cases of syphilis diagnosed at the Faculty of Dentistry, University of the Western Cape (UWC, South Africa), and describe the oral manifestations of syphilis and highlight the role of OHPs in disease management.

## 2. Case Report and Histological Analyses

The four cases described sought treatment at the Department Oral Medicine and Periodontics, Faculty of Dentistry, University of the Western Cape (UWC), South Africa.

### 2.1. Case Report 1

An 18-year-old, otherwise healthy female patient presented complaining of a growth on her tongue with a 4-month duration, which was painful on mastication. She had reported the recent onset of stomach cramps and nausea. Upon further questioning the patient had reported the presence of additional lesions in her anal and vaginal area. The patient is a smoker, currently smoking two cigarettes per day (since the age of 16 years). At the time of the consultation, the patient was managing the discomfort with over the counter analgesics (Ibuprofen and Paracetamol), as well as the topical application of Dynexan 10 g. These medications were provided over the counter as self-medication. The patient had been applying the Dynexan topically, the frequency of application was not disclosed. The dosage and frequency of the analgesics was also not known. Upon further enquiry the patient reported to be sexually active. Extra-oral examination had revealed lymphadenopathy of the right submandibular lymph node.

**Figure 1:**
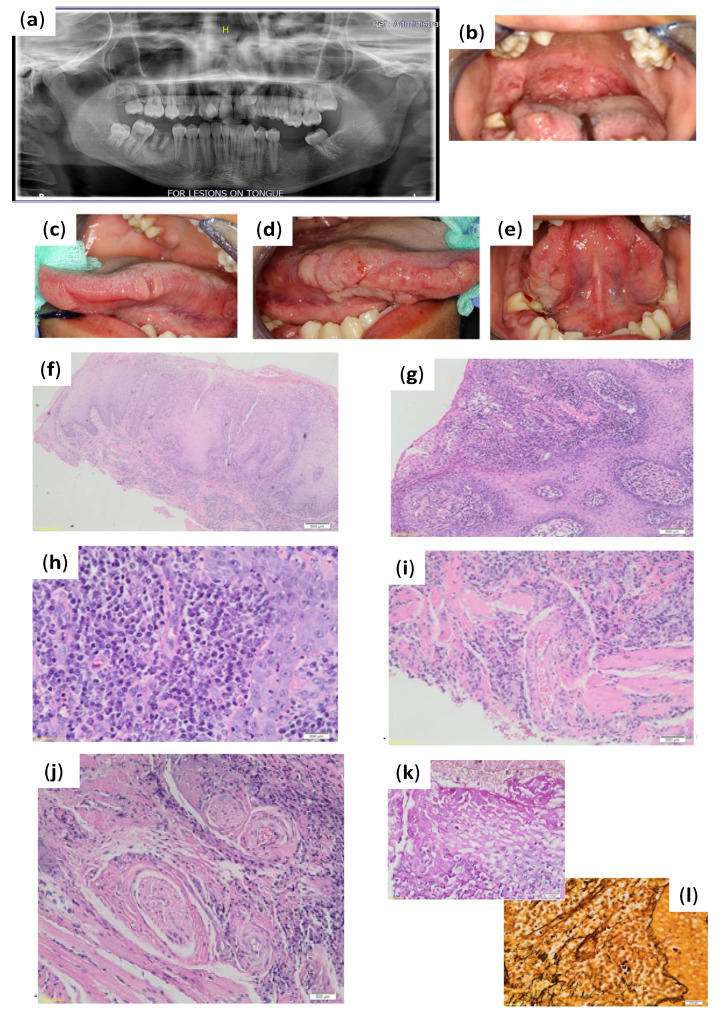
Case 1 clinical presentation of infection and histopathological results from biopsy. (**a**) Pantomograph demonstrating missing teeth 18, 36, and 37. (**b**) Multiple ulcerations involving the entire soft palate and mucous patches involving the hard palate and soft palate. (**c**) Left lateral tongue with two papillary nodules. (**d**) Right lateral tongue with multiple indurated papillary nodules. (**e**) Ventral aspect of tongue demonstrating extension of papillary nodules from the lateral borders of tongue. (**f**) Pseudo-epitheliomatous hyperplasia of the epithelium is evident (hematoxylin and eosin (H&E) magnification ×20). (**g**,**h**) Prominent neutrophil exocytosis, spongiosis and atypia (hematoxylin and eosin (H&E) magnification ×100). (**i**,**j**) The submucosa has a dense perivascular and perineural lymphoplasmacytic infiltrate accompanied by neutrophils and histiocytes (hematoxylin and eosin (H&E) magnification ×200). (**k**) A positive hyphae presence was demonstrated (Periodic Acid Schiff (PAS) magnification ×400). (**l**) The Warthin–Starry stain was positive for spirochaetes. *Treponema Pallidum* noted intra-epithelial and within the lamina propria.

Assessment of the pantomograph (Figure 1a) had revealed missing teeth 18, 36, and 37 (dental charting according to FDI World Dental Federation notation—FDI notation or ISO 3950 notation); root remnant 46 with associated periapical radiolucency; impacted 23, 28, and 48; retained 53; and over-eruption of 26 and 27. Intra-oral examination had revealed multiple ulcerations involving the entire soft palate and mucous patches involving the hard palate, uvula, and right retromolar area (Figure 1b). There were multiple indurated papillary nodules on the tongue involving the tip, the right and left anterior, and mid-portions of the lateral surfaces extending onto the ventral surface (Figure 1c–e).

Based on the history and clinical presentation the differential diagnosis that was considered for this case included bacterial infections (such as tuberculosis and syphilis), deep fungal infections (such as mucormycosis and blastomycosis) and metabolic disease (such as Crohn’s disease).

Special investigations such as serology and incisional biopsy were performed with patient consent. The serologic investigation had included full blood count, differential count, syphilis serology (Rapid Plasma Reagin RPR (BD Macro-Vue RPR Card Test Control Cards, Becton, Dickinson and Company, Franklin Lakes, NJ, USA), and *T. pallidum* antibodies (Elecsys Syphilis assay, Roche Diagnostics, Indianapolis, IN, USA) and HIV serology (HIV-1/2 Ab/Ag ELISA, Elecsys HIV combi PT, Roche Diagnostics, Indianapolis, IN, USA). Incisional biopsies were taken from the right retromolar area and right posterior tongue and sent for histopathological evaluation. Significant findings from the special investigations revealed *T. pallidum* antibodies that were reactive, with RPR reactivity and an RPR titre of 128. The HIV-serology was negative.

The histopathological evaluation had demonstrated pseudo-epitheliomatous hyperplasia (Figure 1f,g) of the epithelium, with prominent neutrophil exocytosis, spongiosis, and atypia induced by inflammation (Figure 1f,g). A dense perivascular and perineural lymphoplasmacytic infiltrate was evident in the submucosa, which was accompanied by neutrophils and histiocytes (Figure 1h,i). Granulomatous inflammation was absent. Immunohistochemistry revealed *T. pallidum* intra-epithelial and within the lamina propria. The Warthin–Starry stain was positive for spirochaetes. There was concomitant candida infection that was confirmed by a positive hyphae presence using the Periodic Acid Schiff (PAS) stain (Figure 1i,j). The diagnosis of secondary syphilis with concomitant candida infection was made. Based on the clinical, histopathological, and serological findings, a definitive diagnosis of syphilis had been determined. The patient was referred to the infectious disease clinic for appropriate management. The patient had expressed concern regarding stigmatization if treated at a facility near her home, as well as fear of her mother knowing the diagnosis. She was then referred to a private hospital for management. The patient was treated with 2.4 MU benzathine benzylpenicillin intramuscular (IM) once weekly for three weeks. The necessary dental treatment required was also arranged. Follow-up with the patient after one month revealed the oral lesions had resolved.

### 2.2. Case Report 2

A 25-year-old, otherwise healthy male had presented with a reportedly painful lesion of the tongue with a 5-month duration. The patient had reported that his wife also had a lesion on her tongue. The patient reported to smoke five cigarettes per day. Upon further enquiry the patient reported to be sexually active.

**Figure 2:**
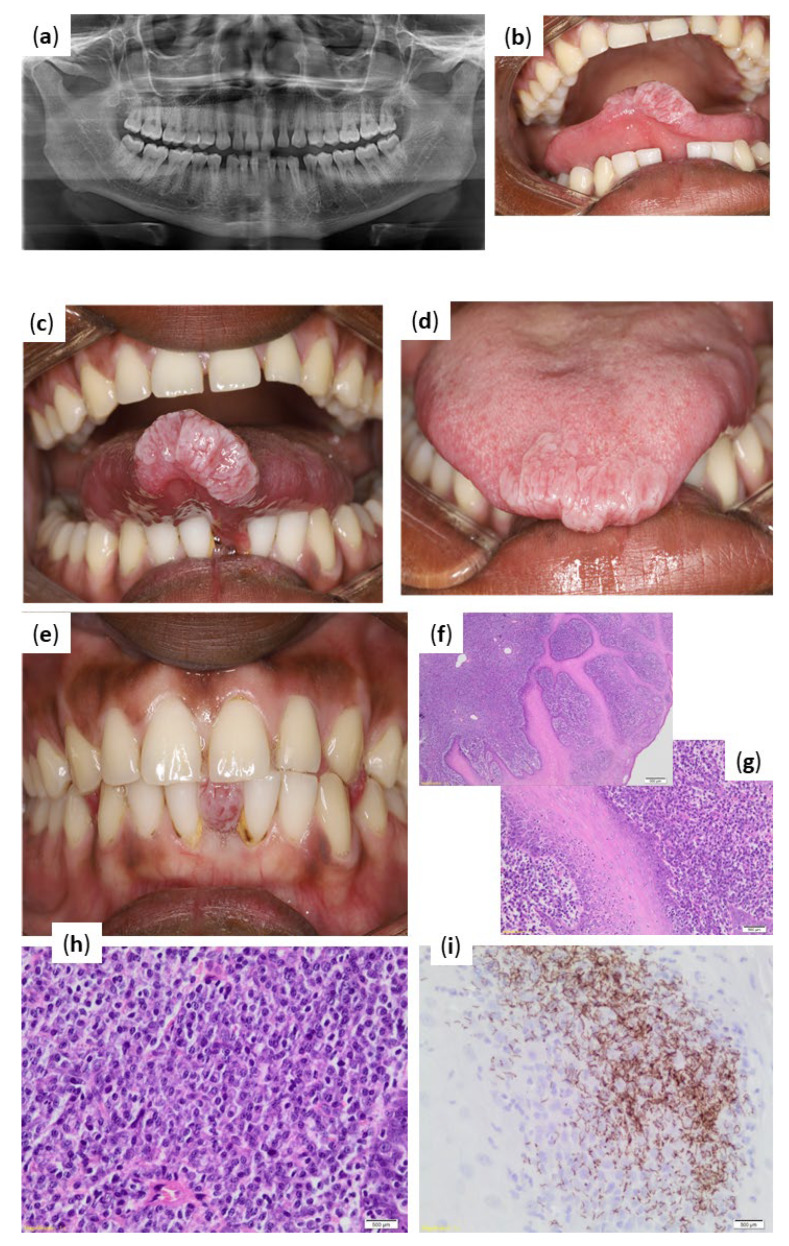
Case 2 clinical presentation of infection and histopathological results from biopsy. (**a**) Pantomograph demonstrating all teeth are present; spacing of teeth in the anterior maxilla and mandible; isolated vertical bone loss on the mesial aspect of 22. (**b**,**c**) Tongue elevated demonstrating ventral aspect with exophytic, well-defined, whitish in colour, lobulated lesion. (**d**) Tongue at rest demonstrating exophytic, whitish, lobulated lesion. (**e**) Protrusion of tongue lesion through diastema between tooth 31 and 41. (**f**) Hyperplastic squamous mucosa with a dense inflammatory infiltrate (hematoxylin and eosin (H&E) magnification ×40). (**g**) Dense submucosal inflammation with plasma cells (hematoxylin and eosin (H&E) magnification ×100). (**h**) Predominantly plasmacytic inflammatory infiltrate (hematoxylin and eosin (H&E) magnification ×400). (**i**) Positive immunohistochemical reaction with *Treponema pallidum*.

The extra-oral examination had revealed no abnormalities. Assessment of the pantomograph had revealed (Figure 2a) all teeth were present; spacing of teeth in the anterior maxilla and mandible; and isolated vertical bone loss on the mesial aspect of 22. The intra-oral examination had revealed an exophytic, well-defined, whitish in colour, firm in consistency, lobulated lesion of approximately 20 × 10 mm in size on the anterior aspect of the tip of the tongue (Figure 2b–d). The patient also presented with a lower diastema, through which the lesion protruded when the patient occluded his teeth (Figure 2e). Based on the history and clinical presentation, the differential diagnosis that was considered for this case included a reactive lesion (such as traumatic fibroma), lesions associated with viral infections such as HPV (squamous papilloma, condyloma acuminata, and verrucous vulgaris), and a possible mesenchymal lesion (such as Schwannoma). Syphilis was initially not considered as a differential diagnosis based on the clinical presentation.

Special investigations such as serology and incisional biopsy were performed with patient consent. The serologic investigation included full blood count, differential count, syphilis serology (Rapid Plasma Reagin RPR (BD Macro-Vue RPR Card Test Control Cards, Becton, Dickinson and Company, Franklin Lakes, NJ, USA), and *T. pallidum* antibodies (Elecsys Syphilis assay, Roche Diagnostics, Indianapolis, IN, USA) and HIV serology (HIV-1/2 Ab/Ag ELISA, Elecsys HIV combi PT, Roche Diagnostics, Indianapolis, Indiana, IN, USA). An incisional biopsy was performed of the tongue lesion and sent for histopathological evaluation.

Histopathological evaluation had shown hyperplastic squamous mucosa with neutrophil exocytosis (Figure 2f) and a dense inflammatory infiltrate comprising largely plasma cells in the submucosa, extending perivascularly (Figure 2g). The polyclonality of the plasma cells was supported by the lack of kappa and lambda restriction on in situ. Predominantly plasmacytic inflammatory infiltrate hybridisation (Figure 2h). Immunohistochemical staining with *T. pallidum* antibody were positive for spirochaetes, which was consistent with a diagnosis of syphilis (Figure 2f).

Significant findings from the special investigations revealed *T. pallidum* antibodies that were reactive, with RPR reactivity and an RPR titre of 64. The HIV-serology was negative.

Based on the clinical, histopathological, and serological findings, a definitive diagnosis of syphilis was made. The patient and his wife were referred to the infectious disease clinic for appropriate management. The patient was treated with 2.4 MU benzathine benzylpenicillin intramuscular (IM) once weekly for three weeks. Telephonic follow-up with the patient after one month revealed the tongue lesion had almost completely resolved.

### 2.3. Case Report 3

A 17-year-old, otherwise healthy female presented complaining of “multiple bumps” on her tongue and the inside of the lips. She had also complained of “dark marks” on her hands and feet, which itch at times. The patient had reported similar dark lesions had developed in her genital area 3 days prior to her consultation with our clinic. The patient had a history of a miscarriage one year ago and had experienced adverse effects with the contraceptive she was prescribed, which has subsequently been discontinued. Upon further enquiry the patient reported to currently be sexually active.

**Figure 3:**
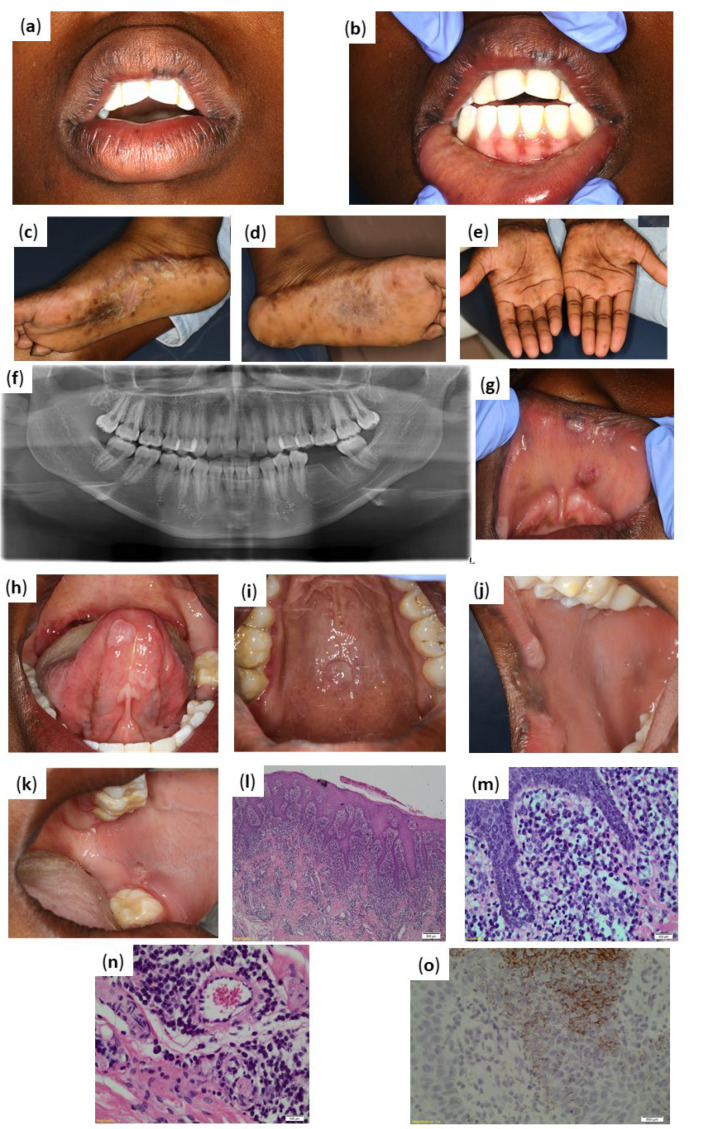
Case 3 clinical presentation of infection and histopathological results from biopsy. (**a**) Lips at rest demonstrating multiple pigmented macules. (**b**) Lips retracted demonstrating pigmented macules not extending beyond the vermillion border of the lip. Generalised physiological pigmentation of inner lower lip and mandibular gingiva noted. (**c**,**d**) Soles of the feet presented with multiple pigmented macules and areas of skin exfoliation. (**e**) Palms of the hands with multiple pigmented macules. (**f**) Pantomograph demonstrating missing teeth 36, 38, and 48; healing extraction socket tooth 36 area. (**g**) Exophytic, sessile soft tissue masses on upper lip. (**h**) Exophytic, sessile soft tissue masses on ventral tongue. (**i**) Localised, exophytic, sessile soft tissue masses posterior palate in midline. (**j**) Localised, whitish soft tissue mass at the right inner aspect of lip in close approximation to the commissure. (**k**) Ulcerated lesion in the buccal mucosa opposite the 37. Areas of leukoedema in left buccal mucosa. (**l**) Hyperplastic squamous mucosa with a dense mixed inflammatory infiltrate (hematoxylin and eosin (H&E) magnification ×20). (**m**) Dense inflammation comprised mainly of plasma cells (hematoxylin and eosin (H&E) magnification ×200). (**n**) Perivascular plasma cells in the submucosa (hematoxylin and eosin (H&E) magnification ×400)**.** (**o**) Positive staining with *Treponema pallidum* on immunohistochemistry.

The extra-oral examination revealed left and right submandibular lymphadenitis. Pigmented macules were noted on the upper and lower lip, not extending beyond the vermillion border (Figure 3b). The palms of the hands and the soles of the feet presented with multiple pigmented macules. Pigmented macules were reported by the patient to be present preceding the oral symptoms. Areas of skin exfoliation were also noted in the centre of the soles of both feet (Figure 3c,d).

Assessment of the pantomograph had revealed (Figure 3f) revealed missing teeth 36, 38, and 48. Healing extraction socket was noted in tooth 36 area. The intra-oral examination had revealed asymptomatic, localised, mucosa coloured with some whiter areas, exophytic and sessile soft tissue masses involving the upper lip (Figure 3g), ventral aspect of the tongue (Figure 3h), and the hard palate (Figure 3i). An ulcerated lesion, approximately 5 mm in diameter was noted in the buccal mucosa opposite the 37, with surroundings areas of leukoedema (Figure 3j,k).

Based on the history and clinical presentation, the differential diagnosis that was considered for this case included lesions associated with viral infections such as HPV (squamous papilloma, condyloma acuminata, verrucous vulgaris, and focal epithelial hyperplasia) and bacterial infections (syphilis).

Special investigations such as serology and excisional biopsy were performed with patient consent. The serologic investigation included full blood count, differential count, syphilis serology (Rapid Plasma Reagin RPR (BD Macro-Vue RPR Card Test Control Cards, Becton, Dickinson and Company, Franklin Lakes, NJ, USA), and *T. pallidum* antibodies (Elecsys Syphilis assay, Roche Diagnostics, Indianapolis, IN, USA) and HIV serology (HIV-1/2 Ab/Ag ELISA, Elecsys HIV combi PT, Roche Diagnostics, Indianapolis, IN, USA). An excisional biopsy was performed of a lesion in the right commissure area.

Significant findings from the special investigations revealed *T. pallidum* antibodies that were reactive, with RPR reactivity and an RPR titre of 32. The HIV-serology was negative.

Histopathological evaluation of the excised commissural lesion had demonstrated hyperplastic squamous mucosa with parakeratosis and spongiosis (Figure 3l). A dense lichenoid lymphoplasmacytic infiltrate consisting predominantly of plasma cells (Figure 3m,n) were noted in the lamina propria, and also extended perineurally and perivascular. No granulomatous inflammation was demonstrated. *T. pallidum* immunohistochemical staining revealed the presence of spirochaetes, which was in keeping with the clinical impression of syphilis (Figure 3o).

Based on the clinical, histopathological, and serological findings, a definitive diagnosis of syphilis was made. The patient was referred to the infectious disease clinic for appropriate management. The patient was treated with 2.4 MU benzathine benzylpenicillin intramuscular (IM) once weekly for three weeks. Follow-up with the patient after one month revealed the oral lesions had resolved.

### 2.4. Case Report 4

A 14-year-old, otherwise healthy female presented with a complaint of an asymptomatic growth on the anterior palate with a duration of 2 months. The patient had reported having multiple “bumps” that manifested on the dorsal surface (at the junction of the anterior 2/3 and posterior 1/3) of the tongue 1 month prior to the growth on the anterior palate. The patient had described these “bumps” as resembling pimples that disappeared after 2 weeks with no treatment. The patient did not smoke nor consume alcohol. Upon further enquiry the patient reported to be sexually active.

**Figure 4:**
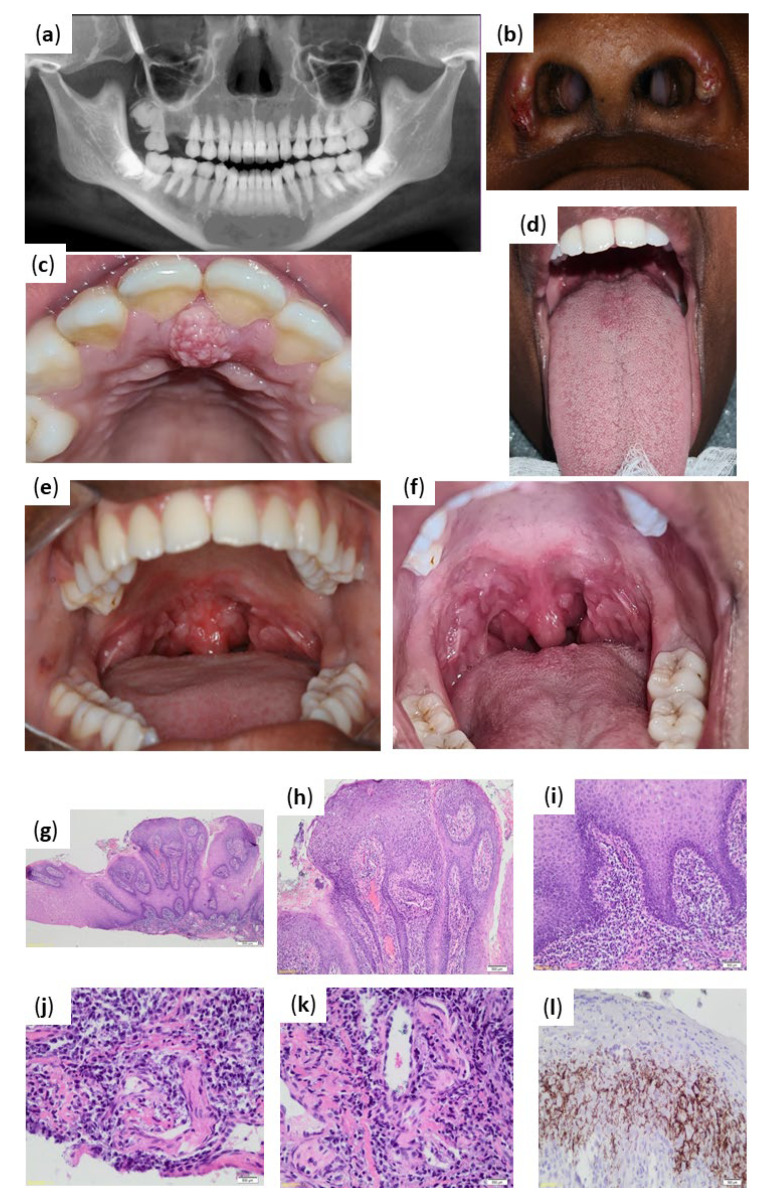
(**a**–**f**) Case 4 clinical presentation of infection and histopathological results from biopsy. (**a**) Pantomograph constructed from CBCT scan demonstrating unerupted 18, 28, 38, and 48; missing 16; and dilacerations of roots of 36 and 37. (**b**) Areas of scab formation on left and right ala of nose. (**c**) Solitary, exophytic, sessile, well-defined, verrucous mass at the position of the incisive papilla. (**d**) Dorsal aspect of tongue with multiple pink papules. (**e**,**f**) Multiple, slightly elevated, white patches surrounded by erythema covering the soft palate, uvula, and tonsillar pillars. (**g**) Papillomatous acanthosis of the squamous mucosa (hematoxylin and eosin (H&E) magnification ×20). (**h**–**i**) Dense submucosal lymphoplasmacytic infiltrate (hematoxylin and eosin (H&E) magnification ×40 and ×100 respectively). (**j**) Perineural plasma cell infiltration (hematoxylin and eosin (H&E) magnification ×400). (**k**) Perivascular plasma cell infiltration (hematoxylin and eosin (H&E) magnification ×400). (**l**) Intraepithelial and submucosal staining with *Treponema pallidum* immunohistochemistry.

Assessment of the pantomograph had revealed (Figure 4a) unerupted 18, 28, 38, and 48; missing 16; and dilacerations of roots of 36 and 37.

The extra-oral examination had revealed left and right submandibular lymphadenitis. Scabs were also noted on the left and right alar of the nose which were superimposed on areas of post inflammatory hyperpigmentation (Figure 4b). The patient had confirmed that no other lesions were present elsewhere on the body.

The intraoral examination had revealed a solitary, firm, exophytic, sessile, well-defined mass at the position of the incisive papilla, with an approximate size of 6 mm × 10 mm. The mass had a mixed white and tissue coloured appearance with a verrucous surface (Figure 4c). The dorsal surface of the tongue had presented with multiple pink papules showing signs of healing (Figure 4d).

Intraoral examination further revealed multiple, asymptomatic, slightly elevated white patches surrounded by erythema covering the soft palate, uvula, and tonsillar pillars. The lesions appeared to coalesce in areas (Figure 4e,f).

Based on the history and clinical presentation, the differential diagnosis that was considered for this case included lesions associated with viral infections such as HPV (squamous papilloma, condyloma acuminata, verrucous vulgaris, and focal epithelial hyperplasia) was identified for the lesion in the incisive papilla area. The differential diagnosis that was considered for the soft palate lesions included bacterial infections (syphilis) and deep fungal infections (such as mucormycosis and blastomycosis).

Special investigations such as serology and excisional biopsy were performed with patient consent. The serologic investigation included full blood count, differential count, syphilis serology (Rapid Plasma Reagin RPR (BD Macro-Vue RPR Card Test Control Cards, Becton, Dickinson and Company, Franklin Lakes, New Jersey, NJ, USA) and *T. pallidum* antibodies (Elecsys Syphilis assay, Roche Diagnostics, Indianapolis, IN, USA), and HIV serology (HIV-1/2 Ab/Ag ELISA, Elecsys HIV combi PT, Roche Diagnostics, Indianapolis, IN, USA). An excisional biopsy was performed of a lesion in the incisive papilla area.

Significant findings from the special investigations revealed a reactive result for *T. pallidum* and the RPR was non-reactive, which was suggestive of a very early or late syphilis infection. The HIV-serology was negative.

Histopathologic evaluation had revealed squamous mucosa with marked papillomatous acanthosis, spongiosis, and leukocyte exocytosis of the epithelium lining the fibrovascular cores (Figure 4g,h). A dense lymphoplasmacytic inflammatory infiltrate was present within the submucosa with a perivascular distribution (Figure 4i). A Periodic Acid-Schiff stain did not demonstrate any fungal elements. Immunohistochemical staining with *T. pallidum* antibody highlighted the presence of spirochaetes in the epithelium and subjacent submucosa. These findings had supported the clinical impression of syphilis (Figure 4j–l).

Based on the clinical, histopathological, and serological findings, a definitive diagnosis of syphilis was made. The patient was referred to the infectious disease clinic for appropriate management. The patient was to be treated with 2.4 MU benzathine benzylpenicillin intramuscular (IM) once weekly for three weeks. The patient had instructed the treating clinician to not inform her parents of the results. The patient had only just commenced treatment upon telephonic follow-up.

## 3. Discussion

Syphilis is a sexually transmitted infection (STI) presenting with skin and mucous membrane lesions [3]. The causative pathogen, *Treponema pallidum* (*T. pallidum*), a spirochete bacteria, may enter an intact mucous membrane, but typically penetrates via micro-abrasions [4]. Upon muco-cutaneous entry, some organisms persist at the inoculation site while others disseminate widely via the lymphatic system [5,6]. The infection will undergo various stages associated with specific oral manifestations.

Syphilis is classified as acquired or congenital. Acquired syphilis involves sexual pathways of transmission (contact with active lesions during oral and genital sex) and by transfusion of infected blood products. Congenital syphilis develops due to vertical transmission from an infected mother to the child [3].

### 3.1. Epidemiology

Syphilis remains an important infection in developed and developing countries due to the associated morbidity and its ability to enhance the transmission of human immunodeficiency virus (HIV) [3]. Syphilitic ulcerations pose a risk factor for the entry of viral HIV. HIV may also affect the natural disease course of syphilis, resulting in patients experiencing more serious complications and relapse after conventional treatment [7].

Based on the estimates in 2016, more than 376 million men and women aged between 15–49 years were infected with one of the four urogenital infections including chlamydia (*Chlamydia trachomatis*), gonorrhoea (*Neisseria gonorrhoea*), syphilis (*Treponema pallidum*), or trichomoniasis (*Trichomonas vaginalis*), which reflects an average of more than one million new infections each day [8]. Globally, there are an estimated 6 million new cases of syphilis each year in individuals in the age groups of 15–49 years. The literature has also reported over 300,000 fetal and neonatal deaths attributed to syphilis [9]. Low and middle income countries demonstrate higher burdens of syphilis infections. High income countries demonstrate concentrated endemics within specific groups of the population, such as men who have sex with men (MSM) [9] (Table 1).

In South Africa, the rates of STIs are of the highest in the world [10,11]. A spectrum model estimation exercise in South Africa (2017), reported active syphilis prevalence at 0.5% for males and 0.97% for females [11].

### 3.2. Oral Manifestations of Syphilis

Syphilis is described as a “great imitator”, due to the vast variation in clinical presentations, presenting a significant challenge to the diagnosing and treating clinician [12].

The manifestations of syphilis are divided into stages (primary, secondary, latent, tertiary, and congenital), each presenting with specific features associated with time and antigen-antibody responses [3,13]. Oral lesions may present at any stage, however are most prominent during the secondary stage.

Primary syphilis is acquired via direct contact with lesions of an infected individual. Upon exposure, incubation lasts approximately 21 days, with the primary lesion developing at the site of inoculation (genital area, anus, or oral cavity) as a painless papule [4,6,12,13,14]. The characteristic sign of primary syphilis is subsequent ulceration of the papule and the development of a chancre. An oral chancre presents as a non-exudating, indurated ulcer, with a raised and erythematous margin. Chancres are generally asymptomatic (unless a secondary infection develops) and heal within 3–6 weeks [4,13]. The primary stage is also characterised by systemic dissemination of the infection.

Approximately 25% of untreated infections will progress to secondary syphilis within 4–6 weeks after primary lesions [4,12,15]. Patients often develop a cutaneous rash presenting as maculopapular or papulosquamous eruptions, commonly affecting the palmar and plantar regions of the hands and feet (as seen in Case 3) [5]. According to a systematic review of oral manifestations of early syphilis in adults, 62.8% of all oral lesions present in the secondary stage of syphilis. During the secondary stage of syphilis, the extra-oral manifestations will be predominately located on the skin, palm, and genital mucosa [14] as reported in Cases 1 (18-year-old female); 3 (17-year-old female); and 4 (14-year-old female).

Oropharyngeal presentations include macules, papules, plaques, ulcers, often a nonspecific pharyngitis, tonsillitis, laryngitis, and lymphadenopathy [16]. The highly infective oral lesions of secondary syphilis rarely develop deep ulceration, showing mucoid exudate and thus the term “mucous patch” is used to describe these lesions. Mucous patches spontaneously regress within 3–12 weeks with or without treatment. The labial commissures may demonstrate split papules and the lateral tongue may develop deep fissures. Additional oral lesions reported include lesions resembling hairy leukoplakia, erythema multiforme, and lichen planus [3,13]. Compared to the lesions of the primary stage, the oral lesions of secondary syphilis are typically associated with discomfort and often present affecting multiple sites. The distribution of lesions is most common on the lips and palate [14]. The uvula is only found to present with lesions in combination with other oral lesions. Uvula involvement is rare (5% of cases), but when it presents the majority of cases (80%) present with mucous patches as reported in Case 1 (18-year-old female) and Case 4 (14-year-old female) [14]. There is no pre-disposition for a specific affected site between genders besides the lips. In men, the upper lip (60%) is more affected than the lower (26.7%) compared to the upper lip of women (45.5%) as reported in Case 3 (17-year-old female). In women, the lower lip and both lips have a similar incidence of 27.3% [14]. When patients present with a single oral lesion, the tongue is the most frequent location. Isolated nodular tongue lesions as demonstrated in Case 2 (25-year-old male) have the least frequent occurrence (9.1%) compared to tongue ulcers and mucous patches (~45.45%, respectively). In patients who present with multiple oral lesions (where the tongue is also affected with a lesion), the tongue will have a nodule in 6.5% of cases compared to an equal distribution for tongue ulcers and mucous patches (~46.75%, respectively) [14].

Patients enter a latent phase once the clinical signs of secondary disease resolve. During latency, individuals will test positive for serologic tests without clinical manifestations [4]. Early latent syphilis is infection of 12 months or less, during which patients may experience muco-cutaneous lesions but are otherwise asymptomatic. Subsequent periods of syphilis (exceeding 12 months and lasting up to 10 years) are referred to as late latent [4,5]. The disease in 60% of untreated patients in the late latent stage follows an asymptomatic course. However, 30–40% progress to late or tertiary disease [13].

The late (or tertiary) phase of infection can be present for 25 years or longer. Tertiary syphilis may manifest in the skin or mucous membranes, central nervous system (tabes dorsalis, general paralysis, insanity, dementia, or seizures) and cardiovascular system (aortitis, aneurysm, and aortic regurgitation) [4,5]. Oral manifestations of tertiary syphilis include oral syphilitic gummas. A gumma is characterised as a large, irregular ulceration with a necrotic base, often associated with extensive destruction of hard and soft tissue in the area, manifesting with palatal perforation. Other infrequent oral presentations may include generalised glossitis with mucosal atrophy. Interstitial glossitis, which develops after the healing of a gumma, is considered to be a premalignant lesion [3,4,13].

Women with untreated syphilis may transmit the disease to their unborn children. Females acutely infected with syphilis during pregnancy are at an increased risk of miscarriage. Some infected infants may present with symptoms already at birth, while others demonstrate symptoms within 2 weeks to 3 months after birth. Early manifestations include desquamative maculopapular rash, rhinitis, vesiculobullous eruptions, radial skin lesions around the oral cavity, fever, skin ulcers, enlarged spleen and liver, jaundice, anaemia, and retardation of fetal growth [3].

Children with untreated syphilis progress to latent and tertiary syphilis. These children present with skeletal damage and developmental abnormalities of the teeth, eyes, ears, and brain [17]. Skeletal defects may be present as a saddle-nose, high arched palate, and frontal bossing of the skull. Hutchinson’s triad may present in late congenital syphilis. Hutchinson’s triad consists of features such as interstitial keratitis (corneal inflammation), cranial nerve VII fallout, peg-shaped permanent incisors, and mulberry multi cusped molars. Late stages of congenital syphilis may present with hydrocephalus, mental retardation, gummas, and neurosyphilis [3,13,18].

### 3.3. Diagnosis

Diagnosis is confirmed based on clinical presentation, histopathologic examination, and serological testing (Table 2). Visual and tactile recognition of lesion morphology and distribution remains a fundamental part of the clinical evaluation by OHPs.

Serological testing, namely nontreponemal (NTP) and treponemal (TP), is a fundamental aspect of establishing a diagnosis, as *T. pallidum* cannot be cultured [19]. The most common combination of serological tests performed to reach a definitive diagnosis are the nontreponemal (such as VDRL and RPR), followed by a treponemal test [20].

### 3.4. Histopathology of Oral Syphilitic Lesions

Histopathologically, a diagnosis of syphilis should be considered in the presence of epithelial hyperplasia, granulomatous or plasma cell chronic inflammation with perivascular distribution, endarteritis, and neuritis [3]. As *T. pallidum* cannot be cultured in vitro, indirect methods are used to detect the pathogen (as described in Table 2). Alternatively, fluorescent stain and dark-field microscopy is routinely used for diagnosis. The endothelial proliferation is seen within small arteries and arterioles, displaying concentric layering of cells with narrowing of the vessel lumen.

Spirochaetes are abundantly observed in microscopic sections from both primary and secondary lesions, with the use of silver stains (Warthin–Starry stain). The spirochaetes do not damage host tissue, thus damage is speculated to be associated with the host response to the bacterium [12]. The necrosis and ulceration demonstrated is thought to be associated with the reduced blood supply.

Microscopically, the highly contagious lesions of secondary and primary syphilis with abundant spirochaetes are indistinguishable [4]. The gumma of tertiary syphilis is seen as granulomatous inflammation with central coagulative necrosis, surrounded by inflammatory infiltrate comprising plasma cells, lymphocytes, activated macrophages, and occasional giant cells, bordered by dense fibrous tissue. In tertiary syphilis, the spirochaetes are extremely sparse or absent, and thus these lesions are less contagious [21].

### 3.5. Management of Syphilis

Currently, the first line therapy for the treatment of primary, secondary, or early latent syphilis includes the use of a single dose of parenteral, long-acting benzathine penicillin G. There are various treatment strategies described in Table 3 and Table 4 for HIV positive and pregnant patients.

Congenital syphilis is best managed by the implementation of preventative measures. The recommendation is thus that all pregnant women undergo serological testing for syphilis. Pregnant women that test positive should be treated with penicillin, with retesting performed at the 28th week and again at delivery. Infants testing positive are to be treated with intravenous aqueous penicillin G or procaine penicillin G for 10 days postpartum [3,13].

### 3.6. Role of Oral Health Professionals

Syphilis holds important implications for the OHPs due to the associated oral manifestations. Due to the apparent re-emergence of this disease, syphilis should be considered as a differential diagnosis when examining patients with ulcerative and erosive oral lesions.

OHPs play an important role in syphilis diagnosis, education, and appropriate referral. All four cases discussed in this article involved patients presenting with a concern regarding oral lesions, thus OHPs were the first point at which medical intervention was requested [3].

OHPs also need to be cautious of the potential transmission of syphilis due to direct contact with lesions, blood, and saliva [3]. Standard infection control measures need to be employed, as not all STIs will present with oral manifestations in all stages of the disease. Even with the onset of syphilis treatment, seroconversion from positive to negative can take months to more than 1 year. Thus, patients undergoing treatment should also be considered as potentially infectious. It is recommended that dental treatment only be provided in the absence of oral lesions. There is also no need to modify the dental treatment plan for a patient with syphilis, as no adverse interactions have been reported between medications used to treat syphilis and drugs commonly used during dental treatment. OHPs are also responsible for reporting a syphilis diagnosis to the appropriate infectious disease authorities [3].

### 3.7. Ethical Considerations and Implications

STIs present a major global public health issue, especially in female populations. The common curable STI’s (chlamydia, gonorrhoea, syphilis, and trichomoniasis) are associated with extensive complications, morbidity, infertility, congenital complications, and poor pregnancy outcomes and increase the risk of HIV acquisition and transmission [22]. The World Health Organization (WHO) has identified improved diagnosis, treatment, and partner services for populations (at high and ongoing risk of acquiring STIs) as key strategies to achieve the global targets to ending STI epidemics by 2030 [8]. Unfortunately, in South Africa, the current standard of care is the management of patients with STI symptoms (syndromic approach), resulting in those that are asymptomatic remaining undiagnosed and untreated. This further increases the risk of transmission of STIs among the population by undiagnosed individuals [2]. Numerous challenges also exist with those patients that are diagnosed, such as patients failing to return for results or treatment and subsequently poor notification rates of partners [23].

Many ethical dilemmas arise for health care workers pertaining to partner notification when a patient is diagnosed with an STI. The ethics surrounding STI control practices involve a balance between respecting individual patient sexual health decisions and protecting the community from STI spread [23].

Effective STI identification and control requires individual treatment, together with partner notification and treatment [24,25]. In all four cases presented in this report, the patients practiced the right to have the responsibility to inform their sexual partners. This creates a scenario of concern, as we as the practitioners are uncertain as to whether partner notification will take place. Currently, the opinion is that the burden of responsibility for health care workers is limited to informing the patient of their STI diagnosis and the importance of notifying their sexual partners. This approach is potentially in conflict with other ethical considerations, such as the harm that may come to an uninformed partner. This highlights the value of health systems implementing contract tracing and notification systems for patients and potential partners with STIs. However, these systems would have to be designed to protect patient autonomy and confidentiality [25,26].

## 4. Conclusions

Syphilis remains a public health concern despite advances in medicine. The growing prevalence of the disease in our immunocompetent population is a reason for concern, indicating a need for public health campaigns with renewed focus on education of the population regarding safe sexual practices. All stages of syphilis can present with oral manifestations and often patients present to OHPs first due to these lesions, as was seen in the four cases described. OHPs thus play an important role in the early recognition and diagnosis of the oral lesions associated with syphilis.

## Figures and Tables

**Table 1:** This table reports on syphilis prevalence across various countries [9].

Country	Syphilis Prevalence
Africa	General prevalence 4–6.5%.
Australia	General increase in cases. 20% increase in MSM cases 2008–2012.
China	Infection rate increased more than three-fold 2005–2014. MSM cases 2010–2014 showed a decrease.
Europe	General prevalence shown a decrease amongst women; increase in infections in men. Men five times more likely to have syphilis than women (2013); 58% of cases were reported among MSM.
USA	Lowest rates of infection recorded 2000–2001. MSM contributed to the increase in the number of primary and secondary cases since 2001. Incidence of syphilis has increased in 2013–2016 amongst all demographic groups. Rates for primary and secondary syphilis higher amongst men (15.6 cases per 100,000 males and 58.1% cases MSM). Incidence of 1.9 cases per 100,000 diagnosed females (2016).

**Table 2:** Methods to establish a diagnosis of syphilis [3,13].

Method	Source for Method	Description	Definitive Diagnosis
Clinical evaluation	History taking and clinical assessment	Clinical findings can suggest a differential diagnosis of syphilis	Definitive diagnosis dependent on microscopic and serological testing
Microscopic assessment: Darkfield Special stains Antibodies DNA/RNA	Fluid and/or surface material collected from lesions Tissue biopsy Tissue biopsy Tissue biopsy	Diagnostic for extra-oral chancre and mucous patches Diagnostic for intra and extra-oral lesions Immunofluorescent antibodies Polymerase chain reaction (PCR) and reverse-transcriptase PCR	Possible for skin and genital lesions Definitive diagnosis Definitive diagnosis Definitive diagnosis
Serological testing: • *Nontreponemal tests (NTT):* Rapid plasma Reagin test (RPR) Venereal disease research laboratory test (VDRL) • *Treponemal tests (TT):* *T. pallidum* particle agglutination assay (TPPA) *T. pallidum* hemagglutination assay (TPHA) Fluorescent treponemal antibody adsorption tests (FTA-ABS) Microhemagglutination assay for antibody to *T. pallidum* (MHA-TP) Treponemal Enzyme Immunoassay(EIA)	Blood, plasma Blood, plasma, saliva, cerebral spinal fluid	Detection of nontreponemal antibodies (cardiolipin) Detection of *T. pallidum* in all stages of infection	Supportive for the diagnosis of syphilis, however specific serological tests required for diagnosis. Quantitative tests that are mainly used for monitoring the status of infection and response to treatment. Qualitative tests to confirm presence of treponemal infection. Patient will test positive for life.

**Table 3:** Summary of management strategies of syphilis in adults (including pregnant women without known HIV infection [3,5,19].

Stage	Treatment	Treatment for Pregnant Woman	Management
Primary, secondary, or early latent syphilis (duration 1 year)	Benzathine penicillin G, 2.4 million Units IM once. If penicillin allergy: Doxycycline 100 mg PO BID for 2 week or tetracycline 500 mg PO QID for 2 weeks. Ceftriaxone 1 g IM or IV for 10–14 days. * Caution: some patients with penicillin allergy are also allergic to ceftriaxone).	Benzathine penicillin G, 2.4 million U IM once (if penicillin allergic, patients should be desensitised and treated with penicillin). Some studies recommend follow up with benzathine penicillin G, 2.4 million U IM administered 1 week after the initial dose. * Caution tetracycline not to be prescribed.	HIV testing. If evidence of neurologic or ophthalmic disease, evaluate for neurosyphilis and do slit-lamp examination Repeat serology and clinical examination at 6 and 12 months for HIV-negative persons. Repeat serology at week 28–32 gestation and at delivery for pregnant women. If symptoms persist or recur, or if a 4-fold increase in RPR or VDRL titer occurs, consider treatment failure or reinfection. Repeat HIV testing, consider LP, and retreat. If RPR or VDRL titers do not fall 4-fold within 6–12 months of treatment, conduct additional clinical and serologic follow-up.
Late latent syphilis or syphilis of unknown duration	Benzathine penicillin G, 2.4 million U IM every week for 3 weeks. If penicillin allergy: Doxycycline,100 mg PO BID for 4 weeks or tetracycline, 500 mg PO QID for 4 weeks.	Benzathine penicillin G, 2.4 million U IM every week for 3 weeks (if penicillin allergy, patients should be desensitised and treated with penicillin).	HIV testing. Examine CSF before treatment if any of the following are present: - Neurologic or ophthalmic signs or symptoms; - Evidence of active tertiary syphilis (e.g., aortitis or gumma); - Treatment failure. Repeat quantitative VDRL or RPR at 6, 12, and 24 months. Repeat serology at 28–32 weeks gestation and at delivery for pregnant women. Patients with a normal CSF examination should be retreated for latent syphilis if: - Serologic titers increase 4-fold; or - An initially high titer (1:32) fails to fall at least 4-fold within 12–24 months or patient develops signs or symptoms consistent with syphilis.
Tertiary syphilis (gumma or cardiovascular syphilis without neurosyphilis)	Benzathine penicillin G, 2.4 million U IM weekly for 3 weeks. Penicillin allergy: follow regimen for late latent syphilis.	Benzathine penicillin G, 2.4 million U IM every week for 3 weeks (if penicillin allergy, patients should be desensitised and treated with penicillin).	HIV testing. Examine CSF.
Neurosyphilis	Aqueous crystalline penicillin G, 3 million to 4 million U IV q4h for 10–14 days. Some studies recommend follow up with benzathine penicillin G, 2.4 million U IM weekly for 3 weeks. * Alternative (if compliance is certain): Procaine penicillin, 2.4 million U IM every day for 10–14 days, plus probenecid, 500 mg PO QID for 10–14 days. Some studies recommend follow up with benzathine penicillin G, 2.4 million U IM weekly for 3 weeks.		HIV testing. Repeat CSF examination every 6 months until CSF cell count is normal.Consider retreatment if cell count has not decreased after 6 months or if CSF is not normal after 2 years.

**Table 4:** Treatment of HIV-positive patients with syphilis [5].

	Treatment	Management
Primary and secondary syphilis	Benzathine penicillin G, 2.4 million U IM once. * Penicillin allergy: manage according to recommendations for HIV-negative patients with primary and secondary syphilis.	Clinical and serologic evaluation 3, 6, 9, 12, and 24 months after therapy. Examine CSF if RPR or VDRL titers fail to show a 4-fold decrease within 6–12 months or there is other evidence of treatment failure. - If CSF normal, retreat with penicillin G, 2.4 million U IM weekly for 3 weeks. - If CSF suggests neurosyphilis, treat for neurosyphilis as in HIV negative patients.
Early latent syphilis	Manage and treat according to recommendations for HIV-negative patients with primary and secondary syphilis. Patients with penicillin allergy whose compliance with therapy or follow-up cannot be ensured should be desensitised and treated with penicillin.	
Late latent syphilis or latent syphilis of unknown duration	Consider Cerebrospinal Fluid (CSF) examination. If CSF normal, give benzathine penicillin G, 2.4 million U IM weekly for 3 weeks. * Penicillin allergy: manage according to recommendations for HIV-negative patients with late latent syphilis or latent syphilis of unknown duration. Patients with penicillin allergy whose compliance with therapy or follow-up cannot be ensured should be desensitised and treated with penicillin. If CSF suggests neurosyphilis, treat for neurosyphilis as in patients that are HIV negative.	Clinical and serologic evaluation 6, 12, 18, and 24 months after therapy. Examine CSF and retreat accordingly if: Clinical symptoms develop or RPR or VDRL titers rise 4-fold at any time or RPR or VDRL titer fails to fall 4-fold between 12 and 24 months.
Neurosyphilis	Management as in HIV negative patients.	

## Data Availability

The datasets generated during and/or analyzed during the current study can be find in the main text.
